# Do Triclosan Sutures Modify the Microbial Diversity of Surgical Site Infections? A Systematic Review and Meta-Analysis

**DOI:** 10.3390/microorganisms10050927

**Published:** 2022-04-28

**Authors:** Frederic C. Daoud, Maïder Coppry, Nicholas Moore, Anne-Marie Rogues

**Affiliations:** 1 INSERM, BPH, U1219, Université de Bordeaux, 33000 Bordeaux, France; maidercoppry@gmail.com (M.C.); nicholas.moore@u-bordeaux.fr (N.M.); anne-marie.rogues@u-bordeaux.fr (A.-M.R.); 2 Pôle de Santé Publique, Service d’Hygiène Hospitalière, CHU Bordeaux, 33000 Bordeaux, France

**Keywords:** surgical site infection, microorganisms, diversity, sutures, triclosan, meta-analysis

## Abstract

Randomised controlled clinical trials (RCTs) report a lower incidence rate of surgical site infections (SSIs) with triclosan sutures (TSs) compared with non-triclosan sutures (NTSs). Do triclosan sutures modify the microbial diversity of culture-confirmed SSIs (ccSSIs)? If so, this would support the association between TS antimicrobial activity and the SSI incidence rate. This prospective systematic literature review (PROSPERO CRD42019125099) was conducted according to PRISMA. RCTs that compared the incidence of SSIs with TSs and NTSs and reported microbial counts from SSI cultures per suture group were eligible. The microbial species were grouped by genus, and the association between genera and sutures was tested. The pooled relative risk (RR) of ccSSIs was also calculated. Twelve RCTs were eligible. No publication bias was identified. The microorganism count was 180 in 124 SSIs with TSs versus 246 in 199 SSIs with NTSs. No significant difference in microbial diversity was found, but statistical power was low for test results to support or challenge the association between the antimicrobial activity of TSs and the reduced rate of SSIs. The RR of the ccSSIs was significant and consistent with comprehensive meta-analyses. The certainty of the pooled RR was moderate.

## 1. Introduction

Surgical site infections (SSI) are diagnosed up to 30 days postoperatively, although some guidelines extend the duration up to one year in prosthetic surgery. SSIs are superficial incisional (skin and subcutaneous tissue), deep incisional (fascia and muscle), and organ/space [1,2]. SSIs may extend across the three domains. SSI surveillance networks report a wide range of incidence rates across operations; e.g., from 0.5% [0.2, 2.7] in prosthetic knee surgery to 10.1% [4.1, 16.9] in laparotomic colon surgery [3].

The precursor of SSI is microbial contamination, and the conceptual relationship of SSI risk has three factors (Formula (1)) [4]:

(1) $$\mathrm{SSI}\;\mathrm{risk}=\frac{\mathrm{bacterial}\;\mathrm{dose}\times \;\mathrm{virulence}}{\mathrm{resistance}\;\mathrm{of}\;\mathrm{the}\;\mathrm{host}\;\mathrm{patient}}\;$$

Virulence refers to disease severity associated with a microorganism. One proposed definition is “the proportion of clinically apparent cases that are severe or fatal” [5]. Virulence varies across microorganisms [6,7,8]. Microorganisms involved in SSI have been reported to originate mainly from the skin, surrounding tissues of the incision, or operated organs with microbial flora such as the bowel [9]. Concerning the bacterial dose, surgical sites contaminated with more than 10^5^/grammes of tissue have a significantly increased risk of SSI [10]. Much lower doses can produce an SSI when foreign material is inside the surgical site, such as sutures; e.g., 100/g of tissue in the case of staphylococci when silk sutures were used [11,12,13].

The guidelines of the World Health Organization (WHO) for SSI prevention conditionally recommend “the use of triclosan-coated sutures to reduce the risk of SSI, independent of the type of surgery” because the quality of the evidence is moderate [14,15]. Triclosan is a broad-spectrum antimicrobial, and in vitro and animal studies have shown that it inhibits microbial colonisation in TSs [16,17,18,19,20,21]. Once implanted, TSs are estimated to display biocidal-level antistaphylococcal activity during the first 4 to 12 h [22]. Therefore, TSs potentially reduce SSI development through the early decrease in bacterial load at the suture surface and the inhibition of suture colonisation.

Prospective randomised controlled clinical trials (RCTs) since 2005 have compared SSI incidence rates with TSs versus NTSs. The most frequently studied TSs have been braided polyglactin 910, with a maximum triclosan load of 472 µg/m; and monofilament polydioxanone and monofilament polyglecaprone, with up to 2360 µg/m [23,24,25].

The pooled relative risks (RRs) and odds ratios (ORs) of comprehensive meta-analyses of RCTs have shown a significantly lower SSI rate with TSs than with NTSs, but most included RCTs were nonsignificant [26,27,28,29,30,31,32]. The meta-analysis with the most data (25 RCTs and 11,957 patients) reported a significant pooled RR of 0.73 [0.65, 0.82] with 88% (22/25) of nonsignificant RCTs [32]. It is unclear whether the significant pooled RR reflected the consequence of TS antimicrobial activity or chance or bias, given the many risk factors of SSIs and the variability in diagnostic criteria [33].

Identifying an expected effect of TS antimicrobial activity on SSIs’ characteristics independent of the pooled RR of the SSIs would support or challenge the association between the use of TSs and the pooled RR.

Microbial susceptibility to triclosan varies by more than 60,000-fold, with a minimum inhibitory concentration (MIC) of 0.016 µg/mL in *Staphylococcus aureus* to more than 1000 µg/mL in *Pseudomonas aeruginosa* and mutant strains of otherwise susceptible species such as *Escherichia coli* or *Klebsiella pneumoniae* [34,35,36,37,38,39,40,41,42,43]. Therefore, one could expect TSs to inhibit microorganisms associated with SSIs in different proportions according to microbial susceptibility to triclosan. A significant difference in microbial diversity of culture-confirmed SSIs (ccSSIs) between TSs and NTSs would be the supportive evidence. One could expect fewer triclosan-susceptible species with TSs, no frequency difference for triclosan-resistant species, or an increase in triclosan-resistant species with TSs due to reduced competition with other species. This systematic literature review (SLR) was performed to test the null hypothesis H0: SSI microbial diversity is not different between TSs and NTSs versus the alternative HA: SSI microbial diversity is different between TSs and NTSs.

## 2. Materials and Methods

### 2.1. Question Framing and Eligibility Criteria

This prospective SLR (PROSPERO CRD42019125099) was conducted according to the Preferred Reporting Items for Systematic Reviews and Meta-Analyses (PRISMA) guidelines [44,45]. The research question was specified according to the Patient, Intervention, Comparison, and Outcome (PICO) framework (Table 1) [46].

### 2.2. Search Strategy

PubMed, Embase, Web of Science, and the Cochrane Library (including CENTRAL) were searched using the following string: “triclosan AND (suture OR sutures OR ligation OR ligations) AND (surgery OR surgeries OR surgical OR operation OR operations) AND ((systematic AND review) OR random* OR RCT OR guide* OR recom* OR meta-analy* OR metaanaly*)” [29,30]. No exclusion filter was applied. The extraction was up to date on 18 August 2021. Appendix A displays the search strategy as implemented in each database (Table A1, Table A2, Table A3 and Table A4).

### 2.3. Eligibility Criteria

Prospective parallel-group RCTs that met the PICO specifications were eligible. Posters, abstracts, communications, and studies that did not report institutional review board or ethics committee approval and patient informed consent were excluded.

### 2.4. Study Selection, Data Extraction, and Risk of Bias Assessment

Two reviewers (F.D. and M.C.) independently conducted the three steps, and the differences were adjudicated by a third reviewer (N.M.). All references were imported into a repository (EndNote X8, Clarivate Analytics, Philadelphia, PA, USA). Eligibility was determined by reading titles, abstracts, and full text. Duplicates were flagged, and multiple publications about the same study were grouped for joint review. Additional studies were identified from the references of RCTs and previous SLRs. Automated queries were used for post hoc verification.

Potentially relevant RCTs were exported to Review Manager (RevMan) 5.4 software (The Cochrane Collaboration, 2020). The individual RCT risk of bias was assessed using the seven items of the built-in risk of bias (RoB) tables [47].

### 2.5. Extracted Data

Data were extracted in standardised tables:

1. Study characteristics: Design, committee approval and informed consent, study registration, statistical methods including power calculation, screening methods, treatment allocation and blinding details, sponsor details, enrollment period and sites, inclusion sites, patient inclusion and exclusion criteria, patient demographics, clinical indication, type of surgery, suture material by suture group, SSI prevention details, and additional patient groups.
2. Detailed patient disposition.
3. Number of patients with a ccSSI by suture type and list of microorganisms per culture or the aggregate count of each microbial designation. When microbial percentages were reported, counts were calculated using the corresponding total number.

### 2.6. Microbial Data Analysis

For descriptive analysis, microbial counts were summed in a spreadsheet according to designation and suture group. The relative frequency of each cell was calculated.

The counts by original designation were then summed according to genus and suture group in a contingency table. Microorganisms that could not be traced to their genus and genera with an expected count of less than n = 5 per cell were excluded from the analysis.

The independence of genera and sutures was tested with Pearson’s chi-squared and Fisher’s exact tests. The significance threshold was *p* < 0.05. The measure of association between genera and sutures was Cramér’s V (0–0.29 weak association, 0.3–0.59 moderate, 0.6–1 strong) [48].

The robustness to sensitivity analysis was tested by iteratively repeating the contingency table analysis with the data of one study removed.

### 2.7. Consistency with Clinical Outcomes

The consistency of microbiological findings with clinical outcomes was assessed by comparing results with the eligible studies’ RRs of ccSSIs (TSs over NTSs). A risk of publication bias was suspected if the funnel plot of the RR was asymmetrical or if Harbord’s test for binary variables was significant (i.e., *p* < 0.05) [49,50].

The heterogeneity of the distribution of the RCTs’ RRs was tested with Cochran’s Q-test (threshold: *p* ≥ 0.05) and the I² statistic, the percentage of variation across the RCTs’ RRs due to heterogeneity rather than chance. The heterogeneity was considered high if I² > 25% [51,52,53,54,55,56]. The robustness of test results was assessed with a sensitivity analysis.

The contingency table analysis, sensitivity analysis, power calculation, and Harbord’s tests were computed in STATA 17 (StataCorp LLC, College Station, TX, USA). The overall bias summary, stratified pooling of RR, heterogeneity analysis, and figure creations were performed in Review Manager 5.3. The risk of bias of the individual RCTs was summarised graphically with Review Manager’s automated table coupled with a forest plot of the RRs. The level of certainty of the pooled RR of the ccSSIs was rated according to GRADE [57].

## 3. Results

### 3.1. Study Identification and Selection

**Figure 1:**
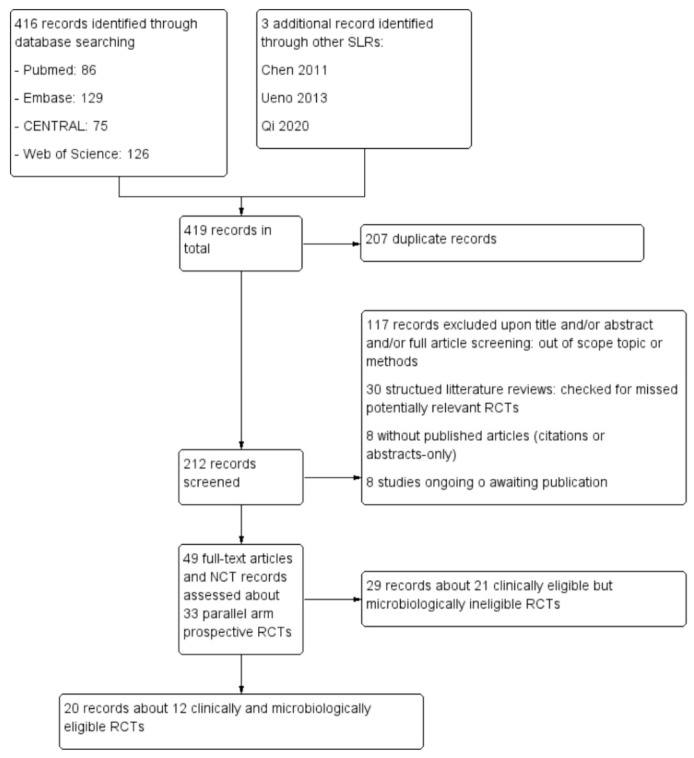
PRISMA flow chart.

A total of 49 records concerning 33 RCTs were in the clinical scope; 20 of them concerning 12 RCTs fulfilled the PICO specifications and were included in the pooled analysis (Figure 1).

### 3.2. Characteristics of Eligible Studies and Risk of Bias

The 12 included studies represented 36% (12/33) of clinically relevant RCTs and included 27% (322/1197) of all SSIs; 25% (3/12) were significant.

The summary of characteristics of the eligible studies showed that half of them were about abdominal surgery (mainly digestive, but also pilonidal and others). The others focused on cardiovascular operations, knee arthroplasty, and neurosurgery (Table 2). Polyglactin sutures were the most frequently compared (83% of the studies), followed by polydioxanone (33%) and polyglecaprone 25 (once). One-third of studies compared associations of TSs.

The counting of microorganisms was straightforward in all but two studies. In Jüstinger 2013, counts were calculated by multiplying the number of ccSSIs by the corresponding percentages of the microorganisms and then rounding decimals to the nearest integer. In Isik 2012, the random allocation ratio was 1 TS to 2 NTSs, thus unbalancing the microbial and SSI counts.

The risk of bias varied significantly across RCTs. The RoB tables of the included RCTs with the supportive information used to rate each item are displayed in Appendix B (Table A5, Table A6, Table A7, Table A8, Table A9, Table A10, Table A11, Table A12, Table A13, Table A14, Table A15 and Table A16).

### 3.3. Microbial Diversity

Microbial diversity consisted of 34 reported species, including remarkable strains (e.g., MRSA) and genera (e.g., *Staphylococcus* spp.) (Table 3). The individual counts were too low to compare the relative frequencies between TSs and NTSs. *E. coli* was the most frequent species, and the only one with a significant RR of 0.58 [0.37, 0.92], with fewer cases in TSs.

The microorganisms were grouped in the contingency table according to eight phylogenetic genera (Table 3). The genera that were excluded due to an expected count of less than five per cell were *Proteus*, *Citrobacter*, *Morganella*, *Corynebacterium*, *Moraxella*, *Serratia*, and *Peptostreptococcus*. Thirty cases designated as polymicrobial or “other bacteria” were also excluded.

The 2-by-8 contingency table had 375 microorganisms, 39% in the TS arm and 61% in NTS (Table 4). The association between genera and sutures was weak (Cramér’s V = 0.11) and nonsignificant (chi-squared *p* = 0.72). The power calculated post hoc was low (1 − β = 0.28). The sensitivity analysis (Supplementary Materials) did not change the conclusions of the overall table and the subtables, so no RCT was identified as a significant cause of bias in the microbial diversity analysis.

The null hypothesis was not rejected.

### 3.4. Clinical Outcomes

**Figure 2:**
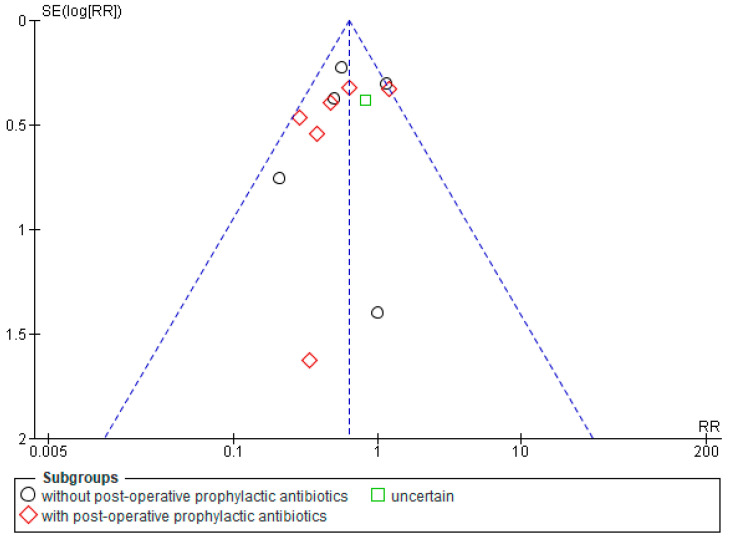
Publication bias analysis—funnel plot.

The funnel plot (Figure 2) showed moderate asymmetry, and Harbord’s test was nonsignificant (*p* = 0.27). Therefore, no publication bias was detected.

**Figure 3:**
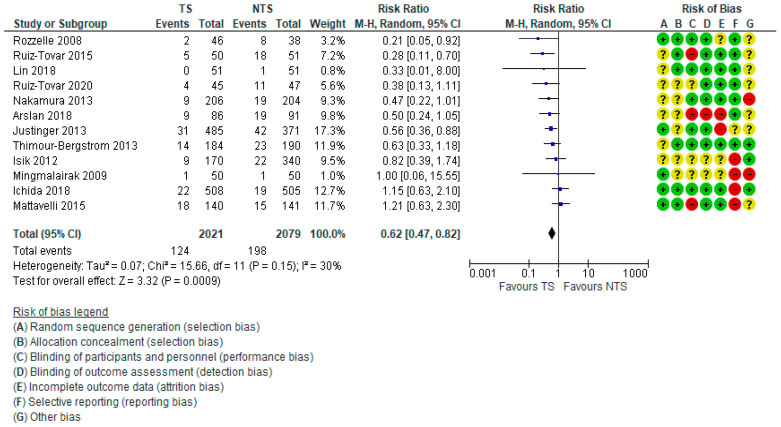
Forest plot—pooled relative risk of ccSSIs and RCTs’ risk of bias [58,59,60,61,62,63,64,65,66,67,68,69].

The meta-analysis of ccSSIs showed a significant RR of 0.62 [0.47, 0.82] favouring TSs. The power calculated *post hoc* was high (1 − β = 0.98), and the overall heterogeneity was moderate (I^2^ =30%, Q-test *p* = 0.15) (Figure 3).

The visual display of RoB for each item and each included RCT is next to the forest plot of the pooled RR (Figure 3).

**Figure 4:**
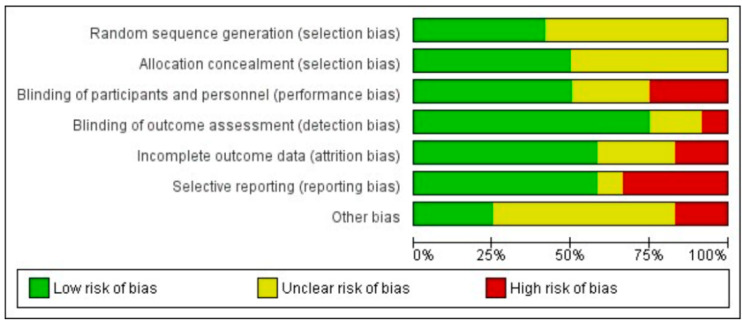
Risk of bias summary of each RoB item as percentages across all included studies.

The average RoB of each item across the included RCTs was low in about half the studies and items combined, and unclear or high in the other half (Figure 4).

The overall RR was robust to the sensitivity analysis (Supplementary Materials).

The level of certainty of the evidence underlying the overall pooled RR of the culture-confirmed SSIs was rated moderate according to GRADE (Table 5).

## 4. Discussion

This review tested if SSI microbial diversity differed between the TS and NTS groups. The protocol assumed that if the TS antimicrobial activity reduced the incidence of SSIs, then SSI cultures’ microbial counts would reflect the microorganism’s triclosan susceptibility.

The contingency table’s independence test was nonsignificant because all eight genera (one per row) reduced the TS column’s total count compared with the NTS column. The ratio of the total microbial count in TSs over NTSs was 0.64. The ratio was 0.65 in *Staphylococcus* (MIC 0.015 to 8 µg/mL), 0.42 in *Escherichia* (0.1 to 0.5 µg/mL), 0.9 in *Enterococcus* (MIC 0.5 to 128 µg/mL; NOTE: MIC > 32 µg/mL is rare), and 0.64 in *Klebsiella* (0.1 to 1 µg/mL), which are usually triclosan-susceptible. The ratio was 0.65 in *Pseudomonas* despite the usual triclosan resistance of most species in human surgery (MIC 100 µg/mL up to ≥1000 µg/mL) [34,35,36,37,38,39,40,41,42,43,70,71,72,73,74]. The sensitivity analysis showed that no RCT contributed enough to the overall dataset for its removal to change the conclusions. That applied to Isik 2012 with a 1:2 allocation ratio; and Jüstinger 2013 with potential inaccuracies in the microbial count.

The absence of a significant difference in the SSIs’ microbial diversity after TSs and NTSs should challenge the association between the difference in the incidence rate of SSIs after TSs and NTSs. However, the statistical power of the chi-squared was low (28%), so the test results could have resulted from chance, and both hypotheses remain plausible.

The power calculation showed that multiplying all cells of the contingency table by 3.5 with the observed proportions would result in a significant chi-squared test result, with *p* = 0.03 and a power of 84%. Such an increase would require a total microbial count of n = 1309. However, such a scenario would still challenge the association between TS and SSI incidence reduction, because the contribution of *Pseudomonas* to the overall lower microbial count in the TS column, with a 0.64 ratio, would be confirmed. Therefore, adding more microbial counts from RCTs would need to show a significant shift of the *Pseudomonas* ratio towards one to demonstrate that *Pseudomonas* were equally frequent in the SSIs of the TS and NTS arms, whereas triclosan-susceptible species remained fewer.

The 12% of excluded culture results were insufficient to bias the contingency table significantly. The designated species are usually intrinsically triclosan-susceptible, and the unspecified cases had an expected 10% triclosan-resistant microorganisms.

No similar study was previously published, so the differences in microbial diversity between the TS and NTS groups of this study could not be compared with other sources.

However, the overall microbial diversity in this study was consistent with the European 2017 SSI surveillance report, in which *Staphylococcus* and *Escherichia* were the most frequent genera, and *P. aeruginosa* represented 4.7% of microorganisms [3]. The microbial diversity was also reasonably consistent with a study of retrieved sutures from SSIs in which *Staphylococcus* was the most frequent genus, and *P. aeruginosa* represented about 5% of microorganisms [75].

The CI of the overall pooled RR of ccSSIs overlapped with the CI of the most comprehensive meta-analysis of RCTs published (Ahmed 2019) [32]. The two studies also agreed in rating the level of evidence as moderate. These similarities suggested that the evidence used here represented the evidence used in Ahmed 2019.

The two limitations of the quality of the evidence in the 12 pooled RCTs; i.e., (1) the minority of significant studies and (2) the uncertain or high risk of bias in about half of the rated points, along with the nonconclusive test of the primary criterion, suggested implementing the WHO conditional guideline with caution. One approach could be making TSs available in routine surgeries for patients with a high risk of SSI or severe SSI complications. Systematically collecting SSI culture details in priority patient groups operated with TSs or NTSs with a minimal clinical dataset incorporated in current surveillance programs would enable an analysis of real-life practice data with evidence from RCTs. That would give those patients a chance to reduce SSI risk with an acceptable risk of adverse suture effects and enable the gathering of evidence to assess the impact of TSs on SSI microbial diversity and ecology. Close monitoring of triclosan-resistant microorganisms such as the *Pseudomonas* genus and mutant strains of usually triclosan-susceptible genera require specific focus.

## 5. Conclusions

This systematic literature review of randomised controlled clinical trials did not show a significant difference in the microbial diversities of surgical site infections after closure using sutures with or without triclosan. However, the amount of evidence was insufficient to support or challenge the relationship between the antimicrobial activities of sutures with triclosan and the incidence rate of surgical site infections.

The meta-analysis of the relative risk of culture-confirmed surgical site infections favoured sutures with triclosan and was consistent with comprehensive meta-analyses. The certainty of the pooled RR was confirmed as moderate.

## Figures and Tables

**Table 1:** PICO specification of the research question.

Item	Specification
Patients	Surgically operated patients
Intervention	Surgical wound closure with any TS
Comparator	Surgical wound closure with any NTS
Outcome	Count of each microorganism isolated in ccSSIs

**Table 2:** Characteristics of eligible studies.

Study	Patients TS, NTS	Enrollment	Type of Surgery	Sutures TS/NTS	Diagnostic Criteria and Follow-Up	ccSSIs/Microorganisms TS, NTS
Ruiz-Tovar 2020 [58]	45 and 50 BTS), 47	4 centers, Spain, 2018–2019	Midline laparotomy, acute abdomen	PDS+ and Stratafix), PDS II	CDC + culture, 30 days	4/4, 11/22
Arslan 2018 [59]	86, 91	1 center, Turkey, 2011–2013	Excision of pilonidal disease	Vicryl+ and PDS+, Vicryl & polypropylene	CDC + culture, 30 days	9/11, 19/22
Ichida 2018 [60]	508, 505	1 center, Japan, 2009–2011	Digestive tract surgery	Vicryl+ and PDS+, Vicryl & PDS II	CDC + culture, 30 days	22/72, 19/59
Lin 2018 [61]	51, 51	1 center, ROC, 2011–2012	Total knee arthroplasty	Vicryl+, Vicryl	Own rules + cultures, 6 months	0/0, 1/1
Mattavelli 2015 [62]	140, 141	4 centers, Italy, 2010–2013	Elective colorectal resection	Vicryl+ and PDS+, Vicryl and PDS II	CDC + culture, 30 days	11/18, 8/13
Ruiz-Tovar 2015 [63]	50, 51	2 centers, Spain, 2007–2013	Fecal peritonitis	Vicryl+, Vicryl	CDC + culture, 60 days	5/5, 18/35
Nakamura 2013 [64]	206, 204	1 center, Japan, 2009–2011	Elective colorectal	Vicryl+, Vicryl	CDC + culture, 30 days	7/12, 13/17
Jüstinger 2013 [65]	485, 371	1 center, Germany, 2009–2011	Laparotomy for various causes	PDS+, PDS II	CDC + culture, 30 days	28/28, 30/30
Thimour-Bergström 2013 [66]	184, 190	1 center, Sweden, 2009–2012	Saphenous vein harvesting, CABG	Vicryl+ and Monocryl+, Vicryl and Monocryl	CDC + culture, 60 days	14/22, 23/29
Isik 2012 [67]	170, 340	1 center, Turkey, 2008–2009	Sternal and saphenous vein harvesting, CABG	Vicryl+, Vicryl	CDC + culture, 30 days	5/5, 9/9
Mingmalairak 2009 [68]	50, 50	1 center, Thailand, 2006–2007	Appendectomy	Vicryl+, Vicryl	Criteria not reported + culture, 30 days	1/1, 1/1
Rozelle 2008 [69]	46, 38	1 center, USA, 2005–2006	CSF shunt in children	Vicryl+, Vicryl	Criteria not reported + culture, 6 months	2/2, 8/8

**Table 3:** Count of microbial species in culture-confirmed SSIs from the 12 RCTs.

Microbial Designations	TS n	TS %	NTS n	NTS %	Total n	Total %
*Staphylococcus aureus*	10	5.6%	26	10.6%	36	8.5%
MRSA	1	0.6%	2	0.8%	3	0.7%
*Coagulase-negative Staphylococcus*	4	2.2%	7	2.8%	11	2.6%
*Staphylococcus epidermidis*	5	2.8%	5	2.0%	10	2.3%
*Staphylococcus* spp.	25	13.9%	29	11.8%	54	12.7%
*Escherichia coli*	22	12.2%	52	21.1%	74	17.4%
*Enterococcus* spp.	18	10.0%	16	6.5%	34	8.0%
*Enterococcus fecalis*	8	4.4%	12	4.9%	20	4.7%
*Enterococcus fecium*	0	0.0%	2	0.8%	2	0.5%
*Enterococcus avium*	1	0.6%	0	0.0%	1	0.2%
*Klebsiella pneumoniae*	13	7.2%	17	6.9%	30	7.0%
*Klebsiella* spp.	4	2.2%	11	4.5%	15	3.5%
*Koxytoca*	1	0.6%	0	0.0%	1	0.2%
*Pseudomonas aeruginosa*	7	3.9%	17	6.9%	24	5.6%
*Pseudomonas* spp.	6	3.3%	3	1.2%	9	2.1%
*Enterobacter* spp.	5	2.8%	7	2.8%	12	2.8%
*Enterobacter cloacae*	4	2.2%	5	2.0%	9	2.1%
*Streptococcus mutans*	2	1.1%	7	2.8%	9	2.1%
*Streptococcus* spp.	3	1.7%	2	0.8%	5	1.2%
*Streptococcus anginosus*	1	0.6%	0	0.0%	1	0.2%
*Bacteroides fragilis*	4	2.2%	6	2.4%	10	2.3%
*Bacteroides* spp.	2	1.1%	1	0.4%	3	0.7%
*Bacteroides ovatus*	0	0.0%	1	0.4%	1	0.2%
*Bacteroides thetaiotaomicron*	0	0.0%	1	0.4%	1	0.2%
*Proteus mirabilis*	2	1.1%	0	0.0%	2	0.5%
*Proteus vulgaris*	2	1.1%	0	0.0%	2	0.5%
*Citrobacter freundii*	0	0.0%	1	0.4%	1	0.2%
*Citrobacter koseri*	1	0.6%	0	0.0%	1	0.2%
*Morganella morganii*	1	0.6%	1	0.4%	2	0.5%
*Peptostreptococcus magnus* (*)	1	0.6%	0	0.0%	1	0.2%
*Corynebacterium* ssp.	0	0.0%	1	0.4%	1	0.2%
*Moraxella catarrhalis*	1	0.6%	0	0.0%	1	0.2%
*Serratia marcescens*	0	0.0%	1	0.4%	1	0.2%
Other bacteria	14	7.8%	11	4.5%	25	5.9%
Polymicrobial	12	6.7%	0	0.0%	12	2.8%
Fungus: *C. Albicans*	0	0.0%	2	0.8%	2	0.5%
TOTAL microorganism count	180	100%	246	100%	426	100%
Culture-confirmed SSIs	124		198		322	
Patients included by authors	2021		2079		4100	

(*) *Finegoldia magna*.

**Table 4:** Count of microbial species in culture-confirmed SSIs from the 12 RCTs.

Genus, n (%)	TS	NTS	Total
*Staphylococcus*	45 (39.47)	69 (60.53)	114 (30.40)
*Escherichia*	22 (29.73)	52 (70.27)	74 (19.73)
*Enterococcus*	27 (47.37)	30 (52.63)	57 (15.20)
*Klebsiella*	18 (39.13)	28 (60.87)	46 (12.27)
*Pseudomonas*	13 (39.39)	20 (60.61)	33 (8.80)
*Enterobacter*	9 (42.86)	12 (57.14)	21 (5.60)
*Streptococcus*	6 (40.00)	9 (60.00)	15 (4.00)
*Bacteroides*	6 (40.00)	9 (60.00)	15 (4.00)
Total	146 (38.93)	229 (61.07)	375 (100)

**Table 5:** GRADE rating of the level of certainty of the evidence supporting the pooled RR of culture-confirmed SSIs.

Certainty Assessment							Summary of Findings				
	Risk of Bias	Inconsistency	Indirectness	Imprecision	Publication Bias	Overall Certainty of Evidence	Study Event Rates (%)		Relative Effect (95% CI)	Anticipated Absolute Effects	
							With Sutures without Triclosan	With Sutures with Triclosan		Risk with Sutures without Triclosan	Risk Difference with Sutures with Triclosan
New outcome (follow up: range 30 days to 365 days; assessed with: clinically and positive culture)											
4100 (12 RCTs)	Serious ^a^	Not serious ^b^	Not serious ^c^	Serious ^d^	None observed	⨁⨁⨁◯ Moderate	198/2079 (9.5%)	124/2021 (6.1%)	RR 0.62 [0.47; 0.82]	95 per 1000	36 fewer per 1000 (s50 to 17 fewer)

CI: confidence interval; RR: relative risk. Explanations: ^a^ Seven studies had insufficient information about random sequence generation and concealment. ^b^ The overall I² was 30%, and heterogeneity assessment with Q-test *p* = 0.15 ^c^ All RCTs had included relevant patients treated who underwent the same type of surgery in the two treatments arms with the compared treatments (TSs versus NTSs). SSIs were culture-confirmed. SSI occurrence was a consequence of multiple factors, but it was the intended clinical effect of TS antimicrobial activity. ^d^ With n_TS = 124 N1 = 2021 and n2 = 198 N1 = 2079, overall power was 98%, which was reasonable to compare the two suture arms. Moreover, only 25% of trials (3/12) were significant.

## Data Availability

Not applicable.

## Supplementary Materials

The following supporting information can be downloaded at: https://www.mdpi.com/article/10.3390/microorganisms10050927/s1. UBordeaux13042022. Supplement. List of excluded randomised clinical trials; Table S1: Source data—microbial count suture treatment arm and per study; Table S2. Sensitivity analysis of the relative risk of culture-confirmed SSIs; Table S3. Sensitivity analysis of the association between genera and suture types.

## Appendix A. Executed Search Strategies

All tables below were set with the same string with the wildcard “*”meaning that the search engine should also retrieve words that are followed with any character. For example: “random*” sets the search engine to retrieve: random, randomised, randomized, randomly, etc. This applies to all four search engines. In Table A1, Pubmed has replaced the “*” with all available explicit words. In Table A2, Table A3 and Table A4, the search engine did not.

**Table A1:** PubMed.

	Query
(“triclosan”[MeSH Terms] OR “triclosan”[All Fields]) AND (“suturability”[All Fields] OR “suturable”[All Fields] OR “sutural”[All Fields] OR “suturation”[All Fields] OR “suture s”[All Fields] OR “sutured”[All Fields] OR “sutures”[MeSH Terms] OR “sutures”[All Fields] OR “suture”[All Fields] OR “suturing”[All Fields] OR (“suturability”[All Fields] OR “suturable”[All Fields] OR “sutural”[All Fields] OR “suturation”[All Fields] OR “suture s”[All Fields] OR “sutured”[All Fields] OR “sutures”[MeSH Terms] OR “sutures”[All Fields] OR “suture”[All Fields] OR “suturing”[All Fields]) OR (“ligate”[All Fields] OR “ligated”[All Fields] OR “ligates”[All Fields] OR “ligating”[All Fields] OR “ligation”[MeSH Terms] OR “ligation”[All Fields] OR “ligations”[All Fields]) OR (“ligate”[All Fields] OR “ligated”[All Fields] OR “ligates”[All Fields] OR “ligating”[All Fields] OR “ligation”[MeSH Terms] OR “ligation”[All Fields] OR “ligations”[All Fields])) AND (“surgery”[MeSH Subheading] OR “surgery”[All Fields] OR “surgical procedures, operative”[MeSH Terms] OR (“surgical”[All Fields] AND “procedures”[All Fields] AND “operative”[All Fields]) OR “operative surgical procedures”[All Fields] OR “general surgery”[MeSH Terms] OR (“general”[All Fields] AND “surgery”[All Fields]) OR “general surgery”[All Fields] OR “surgery s”[All Fields] OR “surgerys”[All Fields] OR “surgeries”[All Fields] OR (“surgery”[MeSH Subheading] OR “surgery”[All Fields] OR “surgical procedures, operative”[MeSH Terms] OR (“surgical”[All Fields] AND “procedures”[All Fields] AND “operative”[All Fields]) OR “operative surgical procedures”[All Fields] OR “general surgery”[MeSH Terms] OR (“general”[All Fields] AND “surgery”[All Fields]) OR “general surgery”[All Fields] OR “surgery s”[All Fields] OR “surgerys”[All Fields] OR “surgeries”[All Fields]) OR (“surgical procedures, operative”[MeSH Terms] OR (“surgical”[All Fields] AND “procedures”[All Fields] AND “operative”[All Fields]) OR “operative surgical procedures”[All Fields] OR “surgical”[All Fields] OR “surgically”[All Fields] OR “surgicals”[All Fields]) OR (“operability”[All Fields] OR “operable”[All Fields] OR “operate”[All Fields] OR “operated”[All Fields] OR “operates”[All Fields] OR “operating”[All Fields] OR “operation s”[All Fields] OR “operational”[All Fields] OR “operative”[All Fields] OR “operatively”[All Fields] OR “operatives”[All Fields] OR “operator”[All Fields] OR “operator s”[All Fields] OR “operators”[All Fields] OR “surgery”[MeSH Subheading] OR “surgery”[All Fields] OR “operations”[All Fields] OR “surgical procedures, operative”[MeSH Terms] OR (“surgical”[All Fields] AND “procedures”[All Fields] AND “operative”[All Fields]) OR “operative surgical procedures”[All Fields] OR “operation”[All Fields]) OR (“operability”[All Fields] OR “operable”[All Fields] OR “operate”[All Fields] OR “operated”[All Fields] OR “operates”[All Fields] OR “operating”[All Fields] OR “operation s”[All Fields] OR “operational”[All Fields] OR “operative”[All Fields] OR “operatively”[All Fields] OR “operatives”[All Fields] OR “operator”[All Fields] OR “operator s”[All Fields] OR “operators”[All Fields] OR “surgery”[MeSH Subheading] OR “surgery”[All Fields] OR “operations”[All Fields] OR “surgical procedures, operative”[MeSH Terms] OR (“surgical”[All Fields] AND “procedures”[All Fields] AND “operative”[All Fields]) OR “operative surgical procedures”[All Fields] OR “operation”[All Fields])) AND (((“classification”[MeSH Terms] OR “classification”[All Fields] OR “systematic”[All Fields] OR “classification”[MeSH Subheading] OR “systematics”[All Fields] OR “systematical”[All Fields] OR “systematically”[All Fields] OR “systematisation”[All Fields] OR “systematise”[All Fields] OR “systematised”[All Fields] OR “systematization”[All Fields] OR “systematizations”[All Fields] OR “systematize”[All Fields] OR “systematized”[All Fields] OR “systematizes”[All Fields] OR “systematizing”[All Fields]) AND (“review”[Publication Type] OR “review literature as topic”[MeSH Terms] OR “review”[All Fields])) OR “random*”[All Fields] OR “RCT”[All Fields] OR “guide*”[All Fields] OR “recom*”[All Fields] OR “meta analy*”[All Fields] OR “metaanaly*”[All Fields]) Translations triclosan: “triclosan”[MeSH Terms] OR “triclosan”[All Fields] suture: “suturability”[All Fields] OR “suturable”[All Fields] OR “sutural”[All Fields] OR “suturation”[All Fields] OR “suture’s”[All Fields] OR “sutured”[All Fields] OR “sutures”[MeSH Terms] OR “sutures”[All Fields] OR “suture”[All Fields] OR “suturing”[All Fields] sutures: “suturability”[All Fields] OR “suturable”[All Fields] OR “sutural”[All Fields] OR “suturation”[All Fields] OR “suture’s”[All Fields] OR “sutured”[All Fields] OR “sutures”[MeSH Terms] OR “sutures”[All Fields] OR “suture”[All Fields] OR “suturing”[All Fields] ligation: “ligate”[All Fields] OR “ligated”[All Fields] OR “ligates”[All Fields] OR “ligating”[All Fields] OR “ligation”[MeSH Terms] OR “ligation”[All Fields] OR “ligations”[All Fields] ligations: “ligate”[All Fields] OR “ligated”[All Fields] OR “ligates”[All Fields] OR “ligating”[All Fields] OR “ligation”[MeSH Terms] OR “ligation”[All Fields] OR “ligations”[All Fields] surgery: “surgery”[Subheading] OR “surgery”[All Fields] OR “surgical procedures, operative”[MeSH Terms] OR (“surgical”[All Fields] AND “procedures”[All Fields] AND “operative”[All Fields]) OR “operative surgical procedures”[All Fields] OR “general surgery”[MeSH Terms] OR (“general”[All Fields] AND “surgery”[All Fields]) OR “general surgery”[All Fields] OR “surgery’s”[All Fields] OR “surgerys”[All Fields] OR “surgeries”[All Fields] surgeries: “surgery”[Subheading] OR “surgery”[All Fields] OR “surgical procedures, operative”[MeSH Terms] OR (“surgical”[All Fields] AND “procedures”[All Fields] AND “operative”[All Fields]) OR “operative surgical procedures”[All Fields] OR “general surgery”[MeSH Terms] OR (“general”[All Fields] AND “surgery”[All Fields]) OR “general surgery”[All Fields] OR “surgery’s”[All Fields] OR “surgerys”[All Fields] OR “surgeries”[All Fields] surgical: “surgical procedures, operative”[MeSH Terms] OR (“surgical”[All Fields] AND “procedures”[All Fields] AND “operative”[All Fields]) OR “operative surgical procedures”[All Fields] OR “surgical”[All Fields] OR “surgically”[All Fields] OR “surgicals”[All Fields] operation: “operability”[All Fields] OR “operable”[All Fields] OR “operate”[All Fields] OR “operated”[All Fields] OR “operates”[All Fields] OR “operating”[All Fields] OR “operation’s”[All Fields] OR “operational”[All Fields] OR “operative”[All Fields] OR “operatively”[All Fields] OR “operatives”[All Fields] OR “operator”[All Fields] OR “operator’s”[All Fields] OR “operators”[All Fields] OR “surgery”[Subheading] OR “surgery”[All Fields] OR “operations”[All Fields] OR “surgical procedures, operative”[MeSH Terms] OR (“surgical”[All Fields] AND “procedures”[All Fields] AND “operative”[All Fields]) OR “operative surgical procedures”[All Fields] OR “operation”[All Fields] operations: “operability”[All Fields] OR “operable”[All Fields] OR “operate”[All Fields] OR “operated”[All Fields] OR “operates”[All Fields] OR “operating”[All Fields] OR “operation’s”[All Fields] OR “operational”[All Fields] OR “operative”[All Fields] OR “operatively”[All Fields] OR “operatives”[All Fields] OR “operator”[All Fields] OR “operator’s”[All Fields] OR “operators”[All Fields] OR “surgery”[Subheading] OR “surgery”[All Fields] OR “operations”[All Fields] OR “surgical procedures, operative”[MeSH Terms] OR (“surgical”[All Fields] AND “procedures”[All Fields] AND “operative”[All Fields]) OR “operative surgical procedures”[All Fields] OR “operation”[All Fields] systematic: “classification”[MeSH Terms] OR “classification”[All Fields] OR “systematic”[All Fields] OR “classification”[Subheading] OR “systematics”[All Fields] OR “systematical”[All Fields] OR “systematically”[All Fields] OR “systematisation”[All Fields] OR “systematise”[All Fields] OR “systematised”[All Fields] OR “systematization”[All Fields] OR “systematizations”[All Fields] OR “systematize”[All Fields] OR “systematized”[All Fields] OR “systematizes”[All Fields] OR “systematizing”[All Fields] review: “review”[Publication Type]. or. “review literature as topic”[MeSH Terms]. or. “review”[All Fields]	

**Table A2:** Embase.

	Query
(‘triclosan’/exp OR triclosan) AND (‘suture’/exp OR suture OR ‘sutures’/exp OR sutures OR ‘ligation’/exp OR ligation OR ligations) AND (‘surgery’/exp OR surgery OR surgeries OR surgical OR ‘operation’/exp OR operation OR operations) AND (systematic AND (‘review’/exp OR review) OR random* OR rct OR guide* OR recom* OR ‘meta analy*’ OR metaanaly*)	

**Table A3:** Web of Science.

	Query
triclosan AND (suture OR sutures OR ligation OR ligations) AND (surgery OR surgeries OR surgical OR operation OR operations) AND ((systematic AND review) OR random* OR RCT OR guide* OR recom* OR meta-analy* OR metaanaly*) (All Fields)	

**Table A4:** Cochrane Library.

	Query
triclosan AND (suture OR sutures OR ligation OR ligations) AND (surgery OR surgeries OR surgical OR operation OR operations) AND ((systematic AND review) OR random* OR RCT OR guide* OR recom* OR meta-analy* OR metaanaly*) in Title Abstract Keyword	

## Appendix B. Risk of Bias (RoB) of Included Studies

**Table A5:** Arslan 2018.

Bias	Author’s Judgement	Support for Judgement
Random sequence generation (selection bias)	Unclear risk	Not reported
Allocation concealment (selection bias)	Unclear risk	Not reported
Blinding of participants and personnel (performance bias)	High risk	No
Blinding of outcome assessment (detection bias)	High risk	No
Incomplete outcome data (attrition bias)	High risk	Patient disposition: no patient lost to follow-up reported. Excluded patients after randomisation and use of allocated sutures due to postoperative administration of antibiotics or use of drains caused a risk of bias.
Selective reporting (reporting bias)	Low risk	Not with respect to ccSSIs
Other bias	Unclear risk	Calculated sample size was not justified with respect to the primary endpoint.

**Table A6:** Ichida 2018.

Bias	Author’s Judgement	Support for Judgement
Random sequence generation (selection bias)	Low risk	Permuted block (size 2) randomisation, although generation process was not described
Allocation concealment (selection bias)	Low risk	Envelope with randomisation code delivered the allocated sutures to the operating room
Blinding of participants and personnel (performance bias)	Low risk	Yes
Blinding of outcome assessment (detection bias)	Low risk	Yes
Incomplete outcome data (attrition bias)	Low risk	Patient disposition: no, as described in details of patient flow
Selective reporting (reporting bias)	High risk	Cultures collected in 22/35 and 9/30 SSIs
Other bias	Low risk	No

**Table A7:** Isik 2012.

Bias	Author’s Judgement	Support for Judgement
Random sequence generation (selection bias)	Unclear risk	Not reported
Allocation concealment (selection bias)	Unclear risk	Not reported
Blinding of participants and personnel (performance bias)	Unclear risk	Reported as double blind, but proedures not described
Blinding of outcome assessment (detection bias)	Unclear risk	Reported as double blind, but proedures not described
Incomplete outcome data (attrition bias)	Unclear risk	Patient disposition: Insufficient details
Selective reporting (reporting bias)	High risk	Fewer data reported about cSSIs than about diagnosed SSIs: sternal TS = 4/170, NTS = 12/328 N.S. (bacteria reported in 4/4 and 8/12); leg TS = 5/142, NTS = 10/160 N.S. (bacteria reported in 2/5 and 2/10)
Other bias	Low risk	No

**Table A8:** Jüstinger 2013.

Bias	Author’s Judgement	Support for Judgement
Random sequence generation (selection bias)	Low risk	Random block sizes of 50 to 100, although the generation process was not described
Allocation concealment (selection bias)	Unclear risk	Reported, but without description
Blinding of participants and personnel (performance bias)	Low risk	Yes
Blinding of outcome assessment (detection bias)	Low risk	Yes
Incomplete outcome data (attrition bias)	High risk	Patient disposition: number of patients excluded after randomisation was much larger than the number of SSIs (111 > 73), especially in the TS group, which had twice as many excluded than the NTS group
Selective reporting (reporting bias)	Unclear risk	The number of patients with culture results and isolated microorganisms compared to the number of SSIs was unclear
Other bias	Unclear risk	Identified bacteria reported as percentages that, when multiplied by the number of SSIs, resulted in numbers with a decimal instead of being integers

**Table A9:** Lin 2018.

Bias	Author’s Judgement	Support for Judgement
Random sequence generation (selection bias)	Unclear risk	Suggested, but mechanisms were not reported
Allocation concealment (selection bias)	Low risk	Sealed envelopes
Blinding of participants and personnel (performance bias)	Low risk	Yes
Blinding of outcome assessment (detection bias)	Low risk	Yes
Incomplete outcome data (attrition bias)	Low risk	Patient disposition: all randomised patients completed study in their group and were included in the analysis
Selective reporting (reporting bias)	Low risk	Not with respect to ccSSIs
Other bias	Unclear risk	Calculated sample size was not justified with respect to the primary endpoint

**Table A10:** Mattavelli 2015.

Bias	Author’s Judgement	Support for Judgement
Random sequence generation (selection bias)	Low risk	Computer-generated list
Allocation concealment (selection bias)	Low risk	Seaed envelopes
Blinding of participants and personnel (performance bias)	High risk	Operators not blinded, although nonoperating staff and patients were blinded
Blinding of outcome assessment (detection bias)	Low risk	Assessor-blinded
Incomplete outcome data (attrition bias)	Low risk	Patient disposition: detailed. Discontinuations explained and not related to SSIs.
Selective reporting (reporting bias)	High risk	Number of cultures less than the number of diagnosed SSIs
Other bias	Unclear risk	Recruited sample size could not be checked against the calculated sample size

**Table A11:** Mingmalairak 2009.

Bias	Author’s Judgement	Support for Judgement
Random sequence generation (selection bias)	Low risk	Random number tables
Allocation concealment (selection bias)	Unclear risk	Insufficiently described
Blinding of participants and personnel (performance bias)	Unclear risk	Insufficiently described
Blinding of outcome assessment (detection bias)	Unclear risk	Insufficiently described
Incomplete outcome data (attrition bias)	Unclear risk	Patient disposition: inconsistencies in flowchart
Selective reporting (reporting bias)	High risk	Inconsistencies in flowchart and ccSSI reporting
Other bias	High risk	Discontinuation after 7.4% of calculated sample size

**Table A12:** Nakamura 2013.

Bias	Author’s Judgement	Support for Judgement
Random sequence generation (selection bias)	Unclear risk	Not reported
Allocation concealment (selection bias)	Unclear risk	Envelope method without further detail
Blinding of participants and personnel (performance bias)	Low risk	No
Blinding of outcome assessment (detection bias)	Low risk	Yes
Incomplete outcome data (attrition bias)	Low risk	Patient disposition: detailed. No losses to follow-up or dropouts
Selective reporting (reporting bias)	Low risk	Not with respect to ccSSIs
Other bias	High risk	Insufficient sample size to reach target power

**Table A13:** Rozzelle 2008.

Bias	Author’s Judgement	Support for Judgement
Random sequence generation (selection bias)	Low risk	Described
Allocation concealment (selection bias)	Low risk	Described
Blinding of participants and personnel (performance bias)	Low risk	Described
Blinding of outcome assessment (detection bias)	Low risk	Described
Incomplete outcome data (attrition bias)	Unclear risk	Patient disposition: no flowchart, but no loss to follow-up reported
Selective reporting (reporting bias)	Low risk	Not with respect to ccSSIs
Other bias	Unclear risk	No sample-size calculation. 37.7% of patients (23/61) were included twice; i.e., 27.4% (23/84) of procedures. The distribution of those 23 dual-inclusions between the two suture groups was not accurately reported, and two observations in the same patient were not statistically independent.

**Table A14:** Ruiz-Tovar 2015.

Bias	Author’s Judgement	Support for Judgement
Random sequence generation (selection bias)	Unclear risk	Not reported
Allocation concealment (selection bias)	Low risk	Sequentially numbered container method
Blinding of participants and personnel (performance bias)	High risk	Randomisation performed by the surgeon without blinding
Blinding of outcome assessment (detection bias)	Low risk	Nurse in charge of diagnosing SSIs was blinded
Incomplete outcome data (attrition bias)	Low risk	Patient disposition flowchart available showed no attrition. Exclusions from SSI incidence comparison were deaths before SSIs.
Selective reporting (reporting bias)	Low risk	Not with respect to ccSSIs
Other bias	Unclear risk	Insufficient information

**Table A15:** Ruiz-Tovar 2020.

Bias	Author’s Judgement	Support for Judgement
Random sequence generation (selection bias)	Unclear risk	Not reported
Allocation concealment (selection bias)	Unclear risk	Not reported
Blinding of participants and personnel (performance bias)	Unclear risk	Operator blinded until the last minute. Operator should have been blinded to the presence of triclosan until the operation was completed.
Blinding of outcome assessment (detection bias)	Low risk	Nurse in charge of SSI diagnosis was blinded as well
Incomplete outcome data (attrition bias)	Low risk	Patient disposition CONSORT flowchart available. No patients lost to follow-up or dropout. Patients excluded due to reoperation or mortality within 30 days were counted. Their exclusions were explainable given the change in risk, and an analysis on an intention-to-treat basis was not performed
Selective reporting (reporting bias)	Low risk	Not with respect to incisional ccSSIs reported, for both incisional and organ/space
Other bias	Unclear risk	Uncertain whether deep and incisional SSIs were in the same patients or different patients. No culture report for deep SSIs.

**Table A16:** Thimour-Bergström 2013.

Bias	Author’s Judgement	Support for Judgement
Random sequence generation (selection bias)	Unclear risk	Not reported, although some details were provided
Allocation concealment (selection bias)	Low risk	Yes
Blinding of participants and personnel (performance bias)	Low risk	Yes
Blinding of outcome assessment (detection bias)	Low risk	Yes
Incomplete outcome data (attrition bias)	Low risk	Patient disposition detailed flowchart showed a small number of patients lost to follow-up or unreachable minor compared to the number of SSIs
Selective reporting (reporting bias)	Low risk	Results reported for all outcome variables described in the methods
Other bias	Low risk	Assuming a one-sided test was planned
