# Supplementary material for: Do Triclosan Sutures Modify the Microbial Diversity of Surgical Site Infections? A Systematic Review and Meta-Analysis
Source: Microorganisms. 2022 Apr 28;10(5):927. doi: 10.3390/microorganisms10050927 (PMC9146332; doi:10.3390/microorganisms10050927)
Supplement: Supplementary file 1 [file microorganisms-10-00927-s001.zip › microorganisms-1705902-supplementary.pdf]

Do Triclosan Sutures Modify the Microbial Diversity of Surgical Site Infections? A Systematic Review and Meta-Analysis

SUPPLEMENTARY MATERIAL

Frederic C. Daoud, MD, MSc, Maïder Coppry, PharmD, PhD, Nicholas Moore, MD, PhD[1] Anne-Marie Rogues, MD, PhD

List of excluded randomised clinical trials

Baracs 2011

Baracs J, Huszar O, Sajjadi SG, Horvath OP. Surgical site infections after abdominal closure in colorectal surgery using triclosan-coated absorbable suture (PDS Plus) vs. uncoated sutures (PDS II): a randomized multicenter study. Surg Infect (Larchmt) 2011;12(6):483-9. [Other: NCT01123616; PubMed: 22142314]

Huszar O, Baracs J, Toth M, Damjanovich L, Kotan R, Lazar G, et al. [Comparison of wound infection rates after colon and rectal surgeries using triclosan-coated or bare sutures -- a multi-center, randomized clinical study]. Magy Seb 2012;65(3):83-91. [DOI: doi.10.1556/MaSeb.65.2012.3.1; PubMed: 22717961]

Chen 2011

Chen S Y, Chen T M, Dai N T, Fu J P, Chang S C, Deng S C, et al. Do antibacterial-coated sutures reduce wound infection in head and neck cancer reconstruction? Eur J Surg Oncol 2011;37(4):300-4. [DOI: doi.10.1016/j.ejso.2011.01.015; PubMed: 21296544]

Ford 2005

Ford H R, Jones P, Gaines B, Reblock K, Simpkins D L. Intraoperative handling and wound healing: controlled clinical trial comparing coated VICRYL plus antibacterial suture (coated polyglactin 910 suture with triclosan) with coated VICRYL suture (coated polyglactin 910 suture). Surg Infect (Larchmt) 2005;6(3):313-21. [DOI: doi.10.1089/sur.2005.6.313; PubMed: 16201941]

Galal 2011

Galal I, El-Hindawy K. Impact of using triclosan-antibacterial sutures on incidence of surgical site infection. Am J Surg 2011;202(2):133-8. [DOI: doi.10.1016/j.amjsurg.2010.06.011; PubMed: 21600552]

Karip 2016

Karip A B, Celik K, Aydin T, Yazicilar H, Iscan Y, Agalar C, et al. Effect of Triclosan-Coated Suture and Antibiotic Prophylaxis on Infection and Recurrence after Karydakis Flap Repair for Pilonidal Disease: A Randomized Parallel-Arm Double-Blinded Clinical Trial. Surg Infect (Larchmt) 2016;17(5):583-8. [DOI: doi.10.1089/sur.2015.207; PubMed: 27383814]

Olmez 2019

Olmez, T. Berkesoglu M. Turkmenoglu O. Colak T., Author, Addresses, Department of Gastrointestinal, Surgery, et al. Effect of Triclosan-Coated Suture on Surgical Site Infection of Abdominal Fascial Closures. Surgical Infections 2019;20(8):658-664. [DOI: 10.1089/sur.2019.052]

Olmez T Colak T, Author Addresses, Department of General Surgery University Hospital of, Mersin Turkey, Correspondence Address, T Olmez Department of General Surgery University Hospital of Mersin, et al. The effect of triclosan coated suture material on surgical site infection of abdominal facial closure. European Surgical Research 2015;55 SUPPL. 1:66-7. [DOI: 10.1089/sur.2019.052]

PROUD 2014

Diener M K, Knebel P, Kieser M, Schuler P, Schiergens T S, Atanassov V, et al. Effectiveness of triclosan-coated PDS Plus versus uncoated PDS II sutures for prevention of surgical site infection after abdominal wall closure: the randomised controlled PROUD trial. Lancet 2014;384(9938):142-52. [DOI: doi.10.1016/s0140-6736(14)60238-5; Other: German clinical study register: DRKS00000390; PubMed: 24718270]

Heger U, Voss S, Knebel P, Doerr-Harim C, Neudecker J, Schuhmacher C, et al. Prevention of abdominal wound infection (PROUD trial, DRKS00000390): study protocol for a randomized controlled trial. Trials 2011;12:245. [DOI: doi.10.1186/1745-6215-12-245; PubMed: 22103965]

Rasic 2011

Rasic Z, Schwarz D, Adam V N, Sever M, Lojo N, Rasic D, et al. Efficacy of antimicrobial triclosan-coated polyglactin 910 (Vicryl* Plus) suture for closure of the abdominal wall after colorectal surgery. Coll Antropol 2011;35(2):439-43. [Other: ISSN 0350-6134; PubMed: 21755716]

Renko 2017

Renko M, Paalanne N, Tapiainen T, Hinkkainen M, Pokka T, Kinnula S, et al. Triclosan-containing sutures versus ordinary sutures for reducing surgical site infections in children: a double-blind, randomised controlled trial. Lancet Infect Dis 2017;17(1):50-7. [DOI: 10.1016/s1473-3099(16)30373-5; Other: NCT01220700; PubMed: 27658562]

Serlo W, Renko M, Paalanne N, Tapaiainen T, Hinkanen M Pokka T, Kinnula S, Sinikumpu Jj, Uhari M. Triclosan-coated sutures in preventing surgical site infection in children: a randomized controlled series. In: Child's nervous system : ChNS : official journal of the International Society for Pediatric Neurosurgery. Vol. 32(10). 2016:1983 (PF-076). [CENTRAL: CN-01742444; DOI: 10.1007/s00381-016-3209-9; Other: NCT01220700]

Roy 2019

Roy, P. K. Kalita P. Lalhlenmawia H. Dutta R. S. Thanzami K. Zothanmawia C. Lalrosangi Pachuau L. Chenkual S., Author, Addresses, Department of Pharmacy, Regional, et al. Comparison of surgical site infection rate between antibacterial coated surgical suture and conventional suture: A randomized controlled single centre study for preventive measure of postoperative infection. International Journal of Pharmaceutical Sciences and Research 2019;10(5):2385-2391. [DOI: 10.13040/IJPSR.0975-8232.10(5).2385-91]

Santos 2019

Santos, Ps, Santos, M, Colafranceschi, As, et al. Effect of Using Triclosan-Impregnated Polyglactin Suture to Prevent Infection of Saphenectomy Wounds in CABG: a Prospective, Double-Blind, Randomized Clinical Trial. Brazilian journal of cardiovascular surgery 2019;34(5):588-595. [DOI: 10.21470/1678-9741-2019-0048; PubMed: 31719010Z]

Seim 2012

Seim B E, Tonnessen T, Woldbaek P R. Triclosan-coated sutures do not reduce leg wound infections after coronary artery bypass grafting. Interact Cardiovasc Thorac Surg 2012;15(3):411-5. [DOI: 10.1093/icvts/ivs266; Other: 22691378]

Soomro 2017

Soomro R, Khurshaidi N, Rahman S S U, Hassan R. Does antibiotic coated polyglactin helps in reducing surgical site infection in clean surgery? 2017;(2):23-26. [CENTRAL: CN-01404947]

Sprowson 2018

Sprowson A P, Jensen C, Parsons N, Partington P, Emmerson K, Carluke I, et al. The effect of triclosan-coated sutures on the rate of surgical site infection after hip and knee arthroplasty: a double-blind randomized controlled trial of 2546 patients. Bone Joint J 2018;100-B(3):296-302. [DOI: 10.1302/0301-620x.100b3.bjj-2017-0247.r1; Other: ISRCTN 17807356; PubMed: 29589500]

Sprowson A P, Jensen C D, Parsons N, Partington P, Emmerson K, Carluke I, et al. The effect of triclosan coated sutures on rate of surgical site infection after hip and knee replacement: a protocol for a double-blind randomised controlled trial. BMC Musculoskelet Disord 2014;15:237. [DOI: 10.1186/1471-2474-15-237; Other: ISRCTN 17807356; PubMed: 25027459]

Steingrimsson 2015

Steingrimsson S, Thimour-Bergstrom L, Roman-Emanuel C, Schersten H, Friberg O, Gudbjartsson T, et al. Triclosan-coated sutures and sternal wound infections: a prospective randomized clinical trial. Eur J Clin Microbiol Infect Dis 2015;34(12):2331-8. [DOI: 10.1007/s10096-015-2485-8; Other: ; Other: NCT01212315; PubMed: 26432552]

Sukeik 2019

Sukeik M, George D, Gabr A, Kallala R, Wilson P, Haddad F S. Randomised controlled trial of triclosan coated vs uncoated sutures in primary hip and knee arthroplasty. World J Orthop 2019;10:268-77. [DOI: 10.5312/wjo.v10.i7.268; PubMed: 31363457]

Haddad Fares University College London Hospital. A randomised controlled trial of triclosan coated sutures in primary total hip and total knee arthroplasty. 2013;http://www.who.int/trialsearch/Trial2.aspx?TrialID=ISRCTN21430045. [CENTRAL: CN-01817028; DOI: org/10.1186/ISRCTN21430045]

Tabrizi 2019

Tabrizi R, Mohajerani H, Bozorgmehr F. Polyglactin 910 suture compared with polyglactin 910 coated with triclosan in dental implant surgery: randomized clinical trial. Int J Oral Maxillofac Surg 2019. [DOI: 10.1016/j.ijom.2019.01.011; Other: NCT03659344; PubMed: 30738711]

Turtiainen 2012

Turtiainen J, Saimanen E I, Makinen K T, Nykanen A I, Venermo M A, Uurto I T, et al. Effect of triclosan-coated sutures on the incidence of surgical wound infection after lower limb revascularization surgery: a randomized controlled trial. World J Surg 2012;36(10):2528-34. [DOI: 10.1007/s00268-012-1655-4; Other: 22618956; Other: NCT01101789]

Turiainen J, Makinin K. Triclosan Coated Suture Wound Closure for Peripheral Vascular Surgery: a Prospective Multicenter Study. www.cochranelibrary.com/central/doi/10.1002/central/CN-01528986/full 2010. [CENTRAL: CN-01528986; DOI: 10.1002/central/CN-01528986; Other: NCT01101789]

Williams 2011

Williams N, Sweetland H, Goyal S, Ivins N, Leaper D J. Randomized trial of antimicrobial-coated sutures to prevent surgical site infection after breast cancer surgery. Surg Infect (Larchmt) 2011;12(6):469-74. [DOI: doi.10.1089/sur.2011.045; Other: NCT008320271; PubMed: 22142317]

Zhang 2011

Zhang Z T, Zhang H W, Fang X D, Wang L M, Li X X, Li Y F, et al. Cosmetic outcome and surgical site infection rates of antibacterial absorbable (Polyglactin 910) suture compared to Chinese silk suture in breast cancer surgery: a randomized pilot research. Chin Med J (Engl) 2011;124(5):719-24. [DOI: 10.3760/cma.j.issn.0366-6999.2011.05.016; Other: NCT00768222; PubMed: 21518565]

Zhuang 2009

Zhuang Chao-ping, Cai Gao-yang, Wang Yong-quan. Comparison of two absorbable sutures in abdominal wall incision. Chinese Journal of Clinical Rehabilitative Tissue Engineering Research 2009 May 21;13(21):4045-8. [Other: CN 21-1539/R CODEN: ZLKHAH; Other: ISSN 1673-8225]

Table S1. Source data - Microbial count suture treatment arm and per study

Group TS	Arslan	Ichida	Isik	Justinger	Mattavelli	Mingmalairak	Nakamura	Rozelle	Ruiz-Tovar2015	Thimour-Bergström	Lin	Ruiz-Tovar2020	total
total	11	72	5	28	18	1	12	2	5	22	0	4	180
Proteus mirabillis	0	0	0	0	0	0	0	0	0	2	0	0	2
Proteus vulgaris	0	0	0	0	2	0	0	0	0	0	0	0	2
Morganella morganii	0	0	0	0	1	0	0	0	0	0	0	0	1
Citrobacter koseri	0	0	0	0	1	0	0	0	0	0	0	0	1
Citrobacter freundii	0	0	0	0	0	0	0	0	0	0	0	0	0
Staph E coli K pneumoniae mix	0	11	0	0	0	0	0	0	0	0	0	0	11
Coryne bacterium ssp	0	0	0	0	0	0	0	0	0	0	0	0	0
Candida albicans	0	0	0	0	0	0	0	0	0	0	0	0	0
Gram+ Gram- mix	0	0	0	0	0	0	0	0	0	1	0	0	1
Moraxella catarrhalis	0	0	0	0	0	0	0	0	0	1	0	0	1
Serratia marcescens	0	0	0	0	0	0	0	0	0	0	0	0	0
MRSA	0	0	0	0	0	0	0	1	0	0	0	0	1
Coag. neg staph	0	0	0	0	0	0	0	1	0	3	0	0	4
S aureus	0	0	0	0	1	1	1	0	0	7	0	0	10
S epidermidis	3	0	1	0	0	0	1	0	0	0	0	0	5
Staphylococcus spp	0	18	0	7	0	0	0	0	0	0	0	0	25
P aeruginosa	0	0	1	0	1	0	0	0	2	3	0	0	7
Pseudomonas spp	0	4	0	2	0	0	0	0	0	0	0	0	6
E coli	6	10	0	0	3	0	0	0	2	0	0	1	22
Bacteroid fragilis	2	0	0	0	1	0	1	0	0	0	0	0	4
Bacteroid ovatus	0	0	0	0	0	0	0	0	0	0	0	0	0
Bacteroid thetaiotao	0	0	0	0	0	0	0	0	0	0	0	0	0
Bacteroid spp	0	0	0	0	0	0	2	0	0	0	0	0	2
K oxytoca	0	0	0	0	1	0	0	0	0	0	0	0	1
Klebsiella pneumoniae	0	10	3	0	0	0	0	0	0	0	0	0	13
Klebsiella spp	0	0	0	0	0	0	0	0	1	1	0	2	4
Enterococcus faecalis	0	0	0	0	2	0	4	0	0	2	0	0	8
Enterococcus faecium	0	0	0	0	0	0	0	0	0	0	0	0	0
Enterococcu ssp	0	8	0	9	0	0	1	0	0	0	0	0	18
E avium	0	0	0	0	1	0	0	0	0	0	0	0	1
Enterobacter cloacae	0	0	0	0	3	0	1	0	0	0	0	0	4
Enterobacter spp	0	4	0	1	0	0	0	0	0	0	0	0	5
Streptococcus anginosus	0	0	0	0	1	0	0	0	0	0	0	0	1
Streptococcus mutans	0	0	0	0	0	0	0	0	0	2	0	0	2
Streptococcus spp	0	0	0	2	0	0	0	0	0	0	0	1	3
Peptostreptococcus magnus	0	0	0	0	0	0	1	0	0	0	0	0	1
Other bactereria	0	7	0	7	0	0	0	0	0	0	0	0	14

Group NTS	Arslan	Ichida	Isik	Justinger	Mattavelli	Mingmalairak	Nakamura	Rozelle	Ruiz-Tovar2015	Thimour-Bergström	Lin	Ruiz-Tovar2020	total
total	22	59	9	30	13	1	17	8	35	29	1	22	246
Proteus mirabillis	0	0	0	0	0	0	0	0	0	0	0	0	0
Proteus vulgaris	0	0	0	0	0	0	0	0	0	0	0	0	0
Morganella morganii	0	0	0	0	1	0	0	0	0	0	0	0	1
Citrobacter koseri	0	0	0	0	0	0	0	0	0	0	0	0	0
Citrobacter freundii	0	0	0	0	0	0	0	0	0	1	0	0	1
Staph E coli K pneumoniae mix	0	0	0	0	0	0	0	0	0	0	0	0	0
Coryne bacterium ssp	0	0	1	0	0	0	0	0	0	0	0	0	1
Candida albicans	0	0	1	0	1	0	0	0	0	0	0	0	2
Gram+ Gram- mix	0	0	0	0	0	0	0	0	0	0	0	0	0
Moraxella catarrhalis	0	0	0	0	0	0	0	0	0	0	0	0	0
Serratia marcescens	0	0	0	0	0	0	0	0	0	1	0	0	1
MRSA	0	0	0	0	0	0	1	1	0	0	0	0	2
Coag. neg staph	0	0	0	0	0	0	0	3	0	4	0	0	7
S aureus	2	0	5	0	1	0	0	3	0	15	0	0	26
S epidermidis	3	0	2	0	0	0	0	0	0	0	0	0	5
Staphylococcus spp	0	19	0	10	0	0	0	0	0	0	0	0	29
P aeruginosa	2	0	0	0	2	1	2	1	9	0	0	0	17
Pseudomonas spp	0	3	0	0	0	0	0	0	0	0	0	0	3
E coli	12	11	0	0	3	0	0	0	16	1	0	9	52
Bacteroid fragilis	3	0	0	0	1	0	2	0	0	0	0	0	6
Bacteroid ovatus	0	0	0	0	0	0	1	0	0	0	0	0	1
Bacteroid thetaiotao	0	0	0	0	0	0	1	0	0	0	0	0	1
Bacteroid spp	0	0	0	0	0	0	1	0	0	0	0	0	1
K oxytoca	0	0	0	0	0	0	0	0	0	0	0	0	0
Klebsiella pneumoniae	0	11	0	0	1	0	0	0	0	0	1	4	17
Klebsiella spp	0	0	0	0	0	0	0	0	5	2	0	4	11
Enterococcus faecalis	0	0	0	0	0	0	4	0	5	3	0	0	12
Enterococcus faecium	0	0	0	0	1	0	1	0	0	0	0	0	2
Enterococcu ssp	0	5	0	10	0	0	1	0	0	0	0	0	16
E avium	0	0	0	0	0	0	0	0	0	0	0	0	0
Enterobacter cloacae	0	0	0	0	2	0	3	0	0	0	0	0	5
Enterobacter spp	0	5	0	2	0	0	0	0	0	0	0	0	7
Streptococcus anginosus	0	0	0	0	0	0	0	0	0	0	0	0	0
Streptococcus mutans	0	0	0	0	0	0	0	0	0	2	0	5	7
Streptococcus spp	0	0	0	2	0	0	0	0	0	0	0	0	2
Peptostreptococcus magnus	0	0	0	0	0	0	0	0	0	0	0	0	0
Other bactereria	0	5	0	6	0	0	0	0	0	0	0	0	11

SENSITIVITY ANALYSES

Table S2. Sensitivity analysis of the relative risk of culture-confirmed SSIs

Study removed	Pooled RR
None	0.62 [0.47, 0.82]
Isik 2012	0.60 [0.44, 0.82]
Arslan 2018	0.63 [0.46, 0.86]
Ichida 2018	0.58 [0.44, 0.75]
Jüstinger 2013	0.62 [0.45, 0.87]
Mingmalairak 2009	0.62 [0.46, 0.82]
Rozzelle 2008	0.65 [0.49, 0.85]
Lin 2018	0.62 [0.47, 0.83]
Mattavelli 2015	0.58 [0.45, 0.75]
Nakamura 2013	0.64 [0.47, 0.86]
Ruiz-Tovar 2015	0.67 [0.51, 0.87]
Ruiz-Tovar 2020	0.64 [0.48, 0.86]
Thimour-Bergström 2013	0.67 [0.49, 0.91]

Table S3. Sensitivity analysis of the association between genera and suture types

Study removed	Total N	Fisher's exact test p	Pearson's Chi² p	Cramer's V
None	375	0.704	0.719	0.1097
Isik 2012	363	0.635	0.646	0.1187
Arslan 2018	342	0.710	0.726	0.1142
Ichida 2018	267	0.558	0.596	0.1438
Jüstinger 2013	330	0.756	0.770	0.1113
Mingmalairak 2009	373	0.700	0.717	0.1103
Rozzelle 2008	365	0.686	0.703	0.1129
Lin 2018	374	0.702	0.718	0.1100
Mattavelli 2015	350	0.732	0.746	0.1106
Nakamura 2013	347	0.657	0.683	0.1178
Ruiz-Tovar 2015	335	0.786	0.786	0.1085
Ruiz-Tovar 2020	349	0.787	0.795	0.1053
Thimour-Bergström 2013	330	0.657	0.664	0.1226
